# Directed Brain Network Analysis for Fatigue Driving Based on EEG Source Signals

**DOI:** 10.3390/e24081093

**Published:** 2022-08-09

**Authors:** Yingmei Qin, Ziyu Hu, Yi Chen, Jing Liu, Lijie Jiang, Yanqiu Che, Chunxiao Han

**Affiliations:** Tianjin Key Laboratory of Information Sensing & Intelligent Control, School of Automation and Electrical Engineering, Tianjin University of Technology and Education, Tianjin 300222, China

**Keywords:** fatigue driving, EEG, current source density, directed network, information integration, causal flow

## Abstract

Fatigue driving is one of the major factors that leads to traffic accidents. Long-term monotonous driving can easily cause a decrease in the driver’s attention and vigilance, manifesting a fatigue effect. This paper proposes a means of revealing the effects of driving fatigue on the brain’s information processing abilities, from the aspect of a directed brain network based on electroencephalogram (EEG) source signals. Based on current source density (CSD) data derived from EEG signals using source analysis, a directed brain network for fatigue driving was constructed by using a directed transfer function. As driving time increased, the average clustering coefficient as well as the average path length gradually increased; meanwhile, global efficiency gradually decreased for most rhythms, suggesting that deep driving fatigue enhances the brain’s local information integration abilities while weakening its global abilities. Furthermore, causal flow analysis showed electrodes with significant differences between the awake state and the driving fatigue state, which were mainly distributed in several areas of the anterior and posterior regions, especially under the theta rhythm. It was also found that the ability of the anterior regions to receive information from the posterior regions became significantly worse in the driving fatigue state. These findings may provide a theoretical basis for revealing the underlying neural mechanisms of driving fatigue.

## 1. Introduction

With improvements in living standards, driving automobiles has become an indispensable part of most people’s lives. Currently, fatigue driving is already been one of the main causes of traffic accidents. Many studies have found that physiological functions of the cerebral cortex change during fatigue driving, including cognitive abilities, information processing, motion control, visual acceptance and timely response [1]. The most commonly used methods to objectively evaluate drivers’ states are based on vehicle behavior characteristics, driver behaviors or drivers’ physiological signals. For the last method, physiological signals, readings from electrooculograms (EOG), electrocardiograms (ECG) and electroencephalograms (EEG) can be used to evaluate brain states, and have become widely applied tools for the recognition of fatigue driving [2,3].

Driving fatigue is defined as a decline in mental ability and efficiency. It has been demonstrated that the underlying neural mechanism of fatigue involves a wide range of brain regions [4]. Many features that are associated with driving fatigue have been extracted from EEG signals, such as time-frequency domain features [5,6], nonlinear features [7,8], entropies [9,10], spatio-temporal features [11,12] and complex network features [13,14].

Complex networks, which can well explain changes in functional connectivity in the brain, have been successfully applied in the study of neurological diseases such as epilepsy and Alzheimer’s disease [15,16]. In the past decade, a lot of research on driving fatigue has also adopted complex network theory [17,18,19,20,21]. However, in most studies, undirected complex networks are mainly used. In fact, the directions of information transfer in the brain are critical for revealing higher-level cognitive mechanisms that underlie different brain states. Hence, it is necessary to explore the connectivity as well as the information flow between different brain regions. Besides, combining EEGs with complex networks to study changes in the brain functions of drivers may provide some objective and effective characteristic indicators and mechanism explanations for driving fatigue. Among them, Granger causality analysis of multivariate time series is widely used in the construction of EEG-based directed networks [21,22,23,24].

Although many remarkable achievements have been made in fields that study fatigue driving based on complex networks, there are still some problems that need to be focused on. On the one hand, some brain networks were constructed using 1 or 2 representative electrodes for each brain region, while about 15 to 20 electrodes were selected as network nodes; however, there are billions of neurons in a brain region. Using more electrodes is needed to improve spatial resolution, which can consequently describe more accurately the dynamic relationships between brain regions. On the other hand, the volume conduction effect and space smearing effect have usually been neglected during EEG pre-processing. Directly collected scalp EEG signals are generated not only from the cerebral cortex where the corresponding electrodes are located, but are also interfered with by multiple brain sources. Therefore, the real brain network cannot be constructed accurately based on the original scalp EEG signals. Source analysis method is used to convert EEGs into current source density data, which can reflect changes in brain activity at the corresponding electrode locations more precisely [25]. EEG source analysis has been successfully applied to improve the classification accuracy of different brain states, including nerve diseases, visual perception, motor imagery and cognition [26,27].

In this paper, we collected EEG signals in the simulated fatigue driving platform, and combined the source analysis method with complex network theory in order to study the dynamic changes in the directed brain network that are induced by the monotonous fatigue driving task under different brain rhythms. The highlights of this paper are as follows: (1) the collected EEG signals were pre-processed by the source analysis to reduce the volume conduction effect; (2) the evolutions and changes in the directed brain network properties for the entire transition from awake to fatigue under different rhythms were fully covered; (3) the changes in the directed information transmission between brain regions affected by driving fatigue were considered. Our findings may unravel the effects of driving fatigue on the brain’s information processing abilities.

## 2. Materials

### 2.1. Subjects

This study included 13 righted-handed undergraduate or graduate students (9 males, 4 females) aged between 20 and 29 years, with normal or corrected vision. All subjects were healthy and had no history of brain neurological diseases. Subjects were told not to take any medicine or drink alcohol, tea or coffee before the experiment. Sufficient sleep the night before the experiment was also required, in order to ensure the stability of the brain states.

### 2.2. Experimental Design

**Figure 1:**
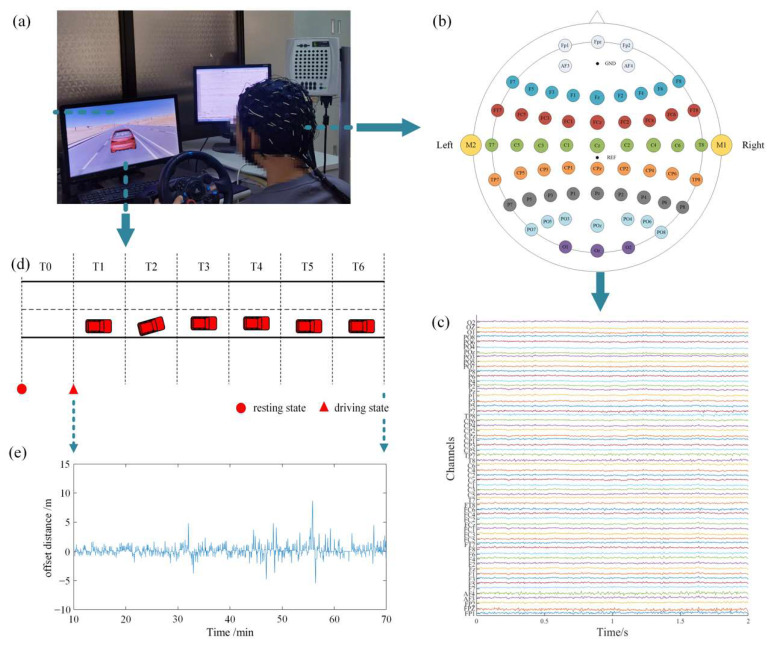
General view of the fatigue driving experiment design. (**a**) Simulated fatigue driving experimental scene; (**b**) electrode locations according to a variant of the international 10–10 system; (**c**) the waveforms of a collected EEG signal segment for one of the subjects; (**d**) experiment flow chart; (**e**) the offset distance vs. time of the vehicle during a fatigue driving experiment for one of the subjects.

A simulated fatigue driving system was built by the Unity 3D game development engine, as shown in Figure 1. The desert was designed as the background of the system to create a monotonous driving environment, making the subjects prone to fatigue. The main task of the experiment was to keep the vehicle in the middle of the left lane while driving. During the experiment, the vehicle was offset randomly and automatically. Whenever there was an offset, subjects needed to adjust the steering wheel in time according to the offset position in order to make the vehicle return to the original lane and continue driving. The offset data were simultaneously collected during the experiment, as shown in Figure 1e. Statistical results show that the offset distance of the vehicle was positively correlated with driving time. As the driving time became longer, the average offset distance became larger. Particularly significant differences were found in driving stages T4 and T5 compared with driving stage T1. The subjects’ attention and vigilance gradually decreased, and they reached a state of fatigue as the experiment went on.

During the experiment, the surrounding environment was maintained in a comfortable state, with moderate temperature, soft light, and no noise interference. The duration of the experiment was 70 min. The first 10 min constituted the resting stage T0, during which the subjects stayed awake quietly without performing any tasks. The remaining 60 min comprised the simulated driving task; it was divided into six driving stages (T1–T6), with each stage lasting 10 min. All subjects were initially trained to be skilled at the driving task, and to be familiar with the software interface. The experiment was conducted in accordance with the rules of the Declaration of Helsinki, and did not violate any morality and ethics. All subjects signed the informed consent form, and had good, cooperative attitudes.

### 2.3. Data Acquisition

In the experiment, EEG signals were acquired using the SynAmps 64-channel Amplifier, Neuroscan. Wet electrodes (Ag/AgCl) were placed according to a variant of the international 10–10 system, as shown in Figure 1b. Sixty-two electrodes were used to record EEG signals, the REF electrode was set as the reference and placed in the vertex of the head between Cz and CPz, while the GND electrode was used as the ground and was located in the middle of the forehead. The sampling rate was 1 kHz, and the electrode impedance was kept below 5 kΩ. For each subject, EEG signals were collected continuously throughout the entire simulated fatigue driving experiment. Based on the experimental task, the collected EEG signals were divided into seven segments that corresponded to stages T0–T6 for further analysis. Each data segment lasted exactly 10 min. In our study, the sliding window technique was used for data analysis. The sliding window length was set to 4 s, while the sliding step applied was 1 s. The data included in one sliding window formed one epoch. Therefore, there were 597 epochs in each stage, and 597×7 epochs overall for each subject, which were used for further data analysis.

## 3. Methods

### 3.1. EEG Pre-Processing

Data pre-processing is particularly essential, since signals usually suffer from interference. In this study, the original EEG signals were first re-referenced to the average of both mastoids, so that there were only 60 electrodes left for the analysis. Then, a high-pass filter with a cut-off frequency of 0.5 Hz was applied to attenuate slow, non-neural electrical potentials; furthermore, a 50 Hz notch filter was used to remove interference that was generated by AC electrical devices. After that, in order to improve the computation efficiency of the following data analysis, EEG signals were down-sampled to 250 Hz by using a polyphase filter. Finally, we adopted the fast independent component analysis (Fast ICA) algorithm [28,29] to remove artifacts from EEG signals, according to the characteristics of different artifacts obtained from observing brain topography, power spectrum and across-trial temporal distributions of ICA components. Two components that were related to blink and oculomotor artifacts were removed for each subject, and the components related to muscle artifacts were removed only if the corresponding artifacts were particularly severe. Most of the pre-processing procedures were performed using an open-source MATLAB toolbox, EEGLAB [29]. Moreover, since the brain dynamics were closely related to the brain rhythms, EEG signals of each electrode were filtered into five typical brain rhythms: delta (0.5–4 Hz), theta (4–8 Hz), alpha (8–13 Hz), beta (13–30 Hz) and gamma (greater than 30 Hz) rhythms.

### 3.2. EEG Source Analysis

**Figure 2:**
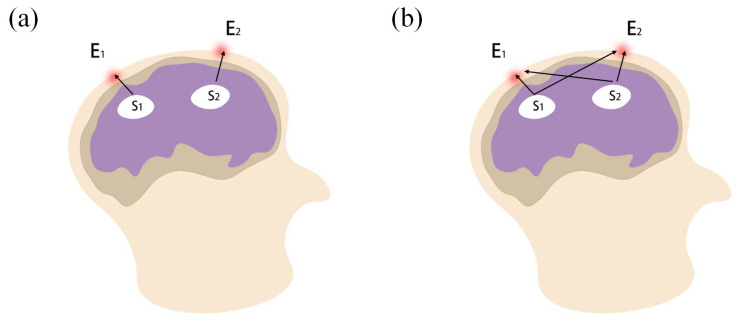
Diagram of the volume conduction effect in EEG measurement. (**a**) EEG measurement without the volume conduction effect; (**b**) EEG measurement with the volume conduction effect.

As a result of the volume conduction effect, collected EEG signals from the scalp reflect the neural electrical activity generated by an ensemble of a large number of pyramidal neurons, not only from the cerebral region where the electrode is located, but also from the surrounding cerebral cortex [30]. For example, in Figure 2, E_1_ and E_2_ represent two electrodes, and S_1_ and S_2_ represent sources below the electrodes. If each electrode only measures the neural electrical activity of the corresponding cerebral cortex below it (Figure 2a), the scalp EEG signals of E_1_ and E_2_ can be directly used to estimate the correlation between sources S_1_ and S_2_. However, both S_1_ and S_2_ contribute to the scalp EEG signals of E_1_ or E_2_ (Figure 2b). Therefore, it is important to obtain the real information that indicates the source activity from the scalp EEG signals. Using EEG source analysis can address the aforementioned problem to a certain extent. It usually uses a spherical model to simulate the head, and applies a Laplacian in order to convert scalp EEG signals into radial currents as the source model [31,32,33].

The current source density (CSD) at any surface point on the sphere is expressed as follows:

(1) $$C\left(E\right)=\overset{n}{\underset{i=1}{\sum }}c_{i}h\left(\cos\left(E,E_{i}\right)\right)$$

where $C\left(E\right)$ is the CSD value at any surface point E on the sphere; $c_{i}$ is a computable constant for a given electrode i that is used to account for a set of surface potentials in the spherical model; $\cos\left(E,E_{i}\right)$ represents the cosine of the angle between the surface point of E and the electrode projection $E_{i}$. The following function $h\left(x\right)$ is defined as the sum of the series.

(2) $$h\left(x\right)=-\frac{1}{4\pi }\overset{\infty }{\underset{n=1}{\sum }}\frac{{\left(2n+1\right)}^{n}}{n^{m}{\left(n+1\right)}^{m}}P_{n}\left(x\right)$$

where m is a constant greater than 1, and $P_{n}$ is the nth Legendre polynomial.

**Figure 3:**
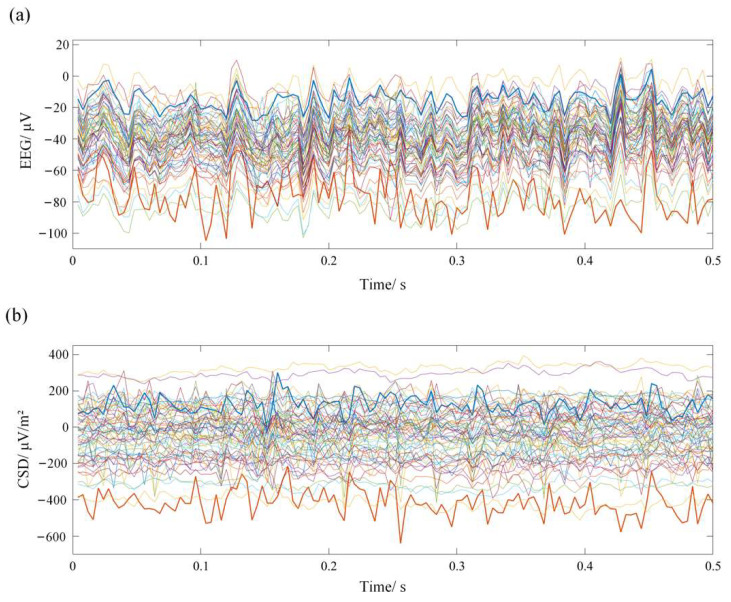
The waveforms of the scalp EEG signals (**a**) and their corresponding CSD data (**b**) resulting from the source analysis.

In this paper, each electrode’s location corresponded to a region of interest (ROI) in the cerebral cortex, which made for a total of 60 ROIs for the following study. With the help of CSD-Toolbox, the scalp EEG signals were converted into standardized CSD data for each ROI. Figure 3 shows the waveforms of a piece of EEG signals, as well as their corresponding CSD data. Although the overall trends were similar, the details were obviously different. Therefore, in our study, we used the CSD data instead of the scalp EEG signals for the following data analysis.

### 3.3. Directed Brain Network Construction

Following the EEG source analysis, CSD data were used to construct a directed brain network. The construction process consisted of the following three steps:

*Step 1*: Select network nodes.

The node in the complex network corresponds to the entity in the complex system, and the entity in this paper corresponds to the electrode. Therefore, there were sixty nodes in the directed brain network, representing different regions of the cerebral cortex.

*Step 2*: Compute directed connectivity between nodes.

The directed connectivity between different nodes was characterized by Directed Transfer Function (DTF) [34,35], which is one of the most widely used Granger causality indexes in the frequency domain based on a multivariate autoregressive (MVAR) model. It provides a robust estimation of the causal information flow from one node to another. The MVAR model is calculated as follows:

(3) $$X\left(n\right)=\overset{p}{\underset{r=1}{\sum }}A_{r}X\left(n-r\right)+W\left(n\right)$$

where p is the order of MVAR model, which is determined by Akaike as well as Bayesian information criteria; $A_{r}$ represents the coefficient matrix; and $W\left(n\right)$ is the Gaussian white noise. The following values can be obtained for a given frequency f:

(4) $$A\left(f\right)=I-\overset{p}{\underset{r=1}{\sum }}A_{r}e^{-2jfr\pi }$$

where I represents the identity matrix. The DTF value of the causal information flow from electrode j to electrode i at frequency f can be solved for using the following formula:

(5) $$DTF\left(i,j,f\right)=\frac{\left|A_{ij}^{-1}\left(f\right)\right|}{\sqrt{\sum_{k}{\left(A_{kj}^{-1}\right)}^{*}\left(f\right)A_{ij}^{-1}\left(f\right)}}$$

where the symbol * represents the complex conjugate transpose of the matrix. Consequently, a 60×60 connectivity matrix can be obtained by observing DTF values between all pairs of electrodes for each epoch under each brain rhythm. Since the connection has direction, the connectivity matrix is asymmetric.

*Step 3*: Choose an optimal threshold and determine edges between nodes.

An edge is a relationship between nodes in a complex network. The edge has direction, indicating a one-way or two-way connection between nodes. Edges in this paper represent directed connections between electrodes. Whether there is a directed edge between two nodes depends on the selected threshold and connectivity strength. A directed edge exists between two nodes if the connectivity strength is greater than the threshold; otherwise, there will be no connection. Then, the directed brain network is obtained, and can be described as a binary adjacency matrix that stores adjacent relationships between nodes. If there is an adjacent relationship between nodes, the corresponding element is 1; otherwise, the element is 0.

In our study, the values of the DTF were used to describe strengths of connectivity. The larger the value of the DTF is, the stronger the connectivity strength. The key issue, next, is to choose the optimal threshold. In this paper, we choose the threshold based on optimal network sparsity, which has been widely used to explore the small-world topology property of the brain network. Network sparsity is defined as the ratio between the number of existing edges and the largest possible number of edges that a constructed network has [36]. Then, the global cost efficiency (GCE) [37] is defined as follows:

(6) GCE=E−S

where S is the network sparsity at a certain threshold, and E is the corresponding global efficiency described in detail in Section 3.4. All possible S values are traversed to compute GCE, and the value of S at the point of maximum GCE is selected as the optimal sparsity of the network. The connectivity strength DTF that makes the network sparsity S reach the optimal value is chosen as the optimal threshold for each sliding window; therefore, different sliding windows may have different thresholds.

### 3.4. Directed Brain Network Parameters

(a) Clustering Coefficient

The clustering coefficient is a commonly used small-world metric to describe the local aggregation ability of a network [21]. It refers to the probability that the adjacents of a node also connect with each other. Assuming that the number of adjacents for node $v_{i}$ in a directed network is $k_{i}$, the maximum possible number of edges between adjacents is $k_{i}\left(k_{i}-1\right)$, while the number of existing edges is $M_{i}$. Then, the clustering coefficient $C_{i}$ of node $v_{i}$ is determined as follows:

(7) $$C_{i}=\frac{M_{i}}{k_{i}\left(k_{i}-1\right)}$$

For a directed network, the average clustering coefficient of multiple electrodes is usually analyzed, which is defined as follows:

(8) $$C=\frac{1}{N}\overset{N}{\underset{i=1}{\sum }}C_{i}$$

where N is the number of nodes in the brain region of interest.

(b) Shortest Path Length

Shortest path length is another small-world index used to represent the global aggregation ability within a complex network [38]. For a directed network, a smaller value of the shortest path length indicates a stronger integration ability between nodes. A directed path with the least number of edges between node i and node j is called the shortest path between these two nodes. In a binary network, the average shortest path length is described as follows:

(9) $$L=\frac{1}{N}\underset{i\in V}{\sum }\frac{\sum_{j\in V,j\neq i}L_{i,j}}{N-1}$$

where N is the number of nodes; $L_{i,j}$ is the number of the edges of the shortest path from node i to node j; and V represents the node set of the network.

(c) Global Efficiency

This efficiency is very closely related to the shortest path length. It can also be used to measure the efficiency of information communication between nodes [39], which is more suitable for a disconnected network. The global efficiency for a directed network is calculated as follows:

(10) $$E=\frac{1}{N\left(N-1\right)}\underset{i,j\in V;i\neq j}{\sum }\frac{1}{L_{i,j}}$$

(d) Causal Flow

In a directed network, the out-degree and the in-degree represent the numbers of edges departing from or pointing to a node, respectively, and can be expressed as follows:

(11) $$k_{i}^{out}=\overset{N}{\underset{j=1}{\sum }}a_{ij}$$

(12) $$k_{i}^{in}=\overset{N}{\underset{j=1}{\sum }}a_{ji}$$

where $a_{ij}$ (or $a_{ji}$) is the element of the binary adjacency matrix with 0 or 1. If the element in the ith row and jth column is 1, it means that there is a directed edge from node i to node j; if the element is 0, the two nodes are not connected. For node i, the out-degree $k_{i}^{out}$ is the sum of the ith row, and the in-degree $k_{i}^{in}$ is the sum of the ith column. Then, the causal flow can be defined as the difference between the out-degree and the in-degree [40]:

(13) $$CF_{i}=k_{i}^{out}-k_{i}^{in}$$

where if $CF_{i}>0$, then node i is considered to mainly affect other nodes; otherwise, node i is mainly affected by other nodes.

### 3.5. Statistical Analysis

A comparison of characteristics between driving stage T0 and stages T1–T6 was carried out using one-way analysis of variance (ANOVA). All characteristics showed a normal distribution according to Kolmogorov–Smirnov tests. In addition, the false discovery rate (FDR) [41], a relatively moderate correction method, was adopted for multiple comparison corrections, which could control the overall type I error and avoid false negatives. In the analysis of clustering coefficients, shortest path lengths and global efficiency, one-way ANOVA was carried out between stages T1–T6 and T0; hence, an FDR correction was performed on six p-values. In the causal flow analysis, one-way ANOVA was carried out on each electrode between stage T4 and T0; hence, an FDR correction was performed on the sixty p-values. The significance level was set at p<0.05.

## 4. Results

### 4.1. Threshold Selection and Directed Network Construction

The threshold of the connectivity strength DFT was determined based on the network sparsity S at which the global cost efficiency GCE reached a maximum. We chose a traversal interval of the network sparsity S ranging from 0.2 to 0.6 with a step of 0.05, which was sufficient to cover the effective range of the small-world properties of the network. We first calculated the GCE according to different S values for each sliding window, and then took the average of the GCEs under all sliding windows over each driving stage for all subjects. Hence, we could obtain the relationship between S and the average GCE during different driving stages under different rhythms for all subjects.

**Figure 4:**
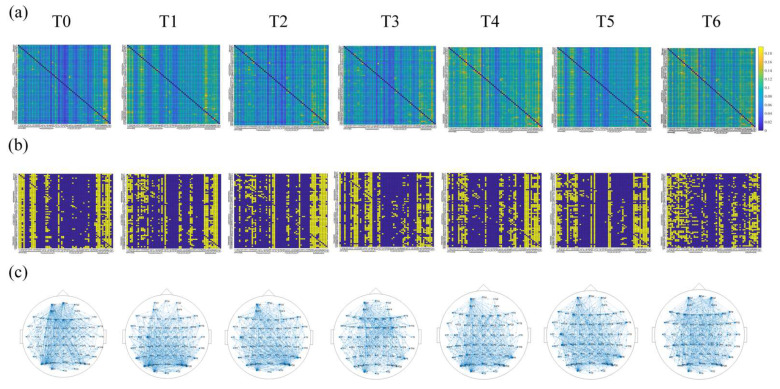
Directed brain networks constructed by the DTF in different driving stages under the theta rhythm for one subject. (**a**) The color map of the connectivity matrix of the directed brain network; (**b**) the color map of the binary adjacency matrix of the directed brain network, where the vertical axis and the horizontal axis represent the corresponding electrodes; blue indicates the value 1 and yellow indicates the value 0; (**c**) the connections diagram of the directed brain network. The optimal network sparsity S  is set as 0.3 here.

Table 1 shows the values of GCE corresponding to different S during different driving stages under the theta rhythm. When S is ~0.3, the GCE value is generally a maximum; similar results were also achieved for other rhythms. Therefore, we chose 0.3 as the optimal network sparsity; that is, for each sliding window, if the connectivity strength DFT was in the top 30%, there would be a directed edge between the corresponding two nodes. The corresponding element in the binary adjacency matrix was set as 1; otherwise, there would be no directed edge, and the corresponding element was set as 0. Finally, the directed brain network was uniquely constructed for each sliding window. Figure 4 shows the color map of the connectivity matrix, the binary adjacency matrix, and the corresponding directed brain network connections diagram during different driving stages under the theta rhythm for one subject. Intuitively, the network topology indeed changed as the driving time went on.

### 4.2. Information Integration Ability Analysis

In order to investigate the effect of driving fatigue on the directed brain network’s ability to integrate information, we studied the small-world property of the brain network under different brain rhythms in terms of the clustering coefficient C, shortest path length L and global efficiency E.

(a) Clustering Coefficient Analysis

**Figure 5:**
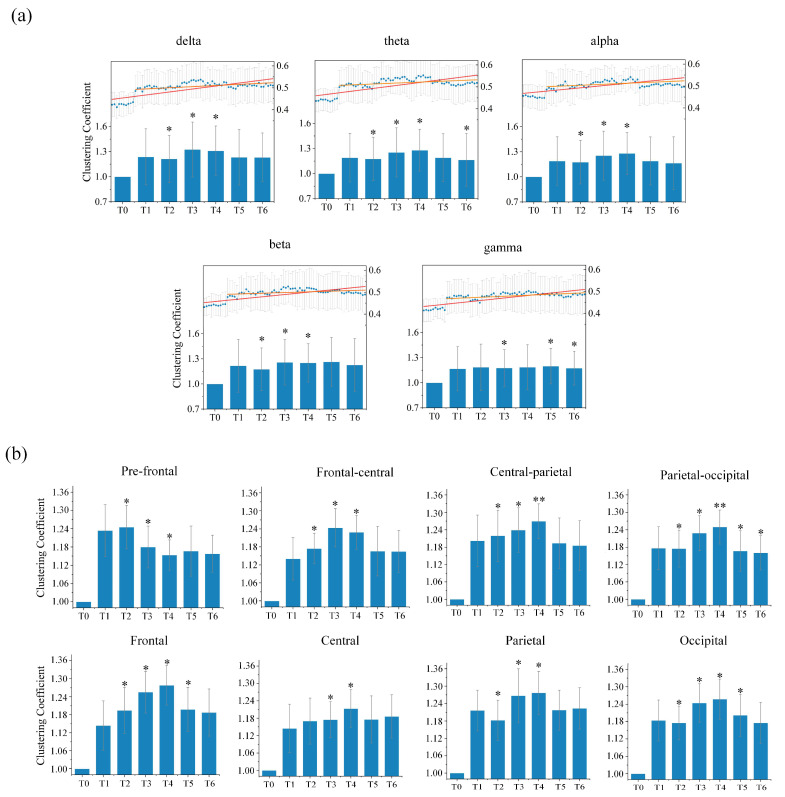
The average clustering coefficients during driving stages T0–T6 for all subjects. (**a**) The average clustering coefficients from the directed brain network under five different rhythms; (**b**) The average clustering coefficients of different brain regions from the directed brain network under theta rhythm. Histograms represent relative changes from the resting stage T0. The blue points in the histogram are the average clustering coefficients calculated from all sliding windows in one minute. The red line is the fitting line for all blue points over driving stages T0–T6, and the orange line is the fitting line for all blue points over driving stages T1–T6. * and ** indicate significant differences compared to resting stage T0 (one-way ANOVA followed by FDR correction; * stands for p<0.05 and ** stands for p<0.01 ).

In order to measure changes in the local information integration ability of the directed brain network during the driving task, we calculated the clustering coefficient for each epoch under different rhythms during different driving stages for all subjects. The results of the average clustering coefficient of the entire directed brain network under five different rhythms for seven stages for all subjects are shown in Figure 5a. As the driving time increased, for the delta, theta, alpha and beta rhythms, the clustering coefficient initially presented an upward trend, reached its highest in stage T3 or T4, and then gradually decreased to a certain extent. However, the gamma rhythm did not change much with increases in driving time. Statistical analysis of the average clustering coefficient was performed in order to assess whether there was a significant difference in driving stages T1–T6 compared to the resting stage T0. It was observed that the delta, theta, alpha and beta rhythms were all significantly different in stages T2, T3 and T4. Moreover, the theta rhythm in stage T6, in addition to the gamma rhythm in stages T3, T5 and T6, were also significantly different. This rising trend was particularly obvious in the low-frequency rhythms. The above results further confirm that changes in local information integration in the brain were related to driving fatigue.

We further studied changes in the average clustering coefficients from each brain region under each rhythm for all subjects. Overall, the average clustering coefficient had a positive correlation with the driving time for most of the brain regions under low-frequency rhythms, indicating that the nodes in most of the directed brain network regions tend to agglomerate, and that its ability to integrate local information is enhanced by driving fatigue. Only the results under the theta rhythm, with the most obvious changes and the most significant differences for the whole brain, are provided, as shown in Figure 5b. As the driving time increased, the average clustering coefficients for each brain region in stages T1–T6 all became higher than those for stage T0. Most brain regions showed changing patterns that were similar to those in the whole brain. In particular, we also found another kind of changing trend in the pre-frontal region. Although the average clustering coefficient was larger in driving stages T1–T6 than the one in resting stage T0, it shows a downward trend with increases in driving time, suggesting that driving for long durations will gradually reduce local information integration abilities in the pre-frontal region. Statistical analysis between the resting stage T0 and driving stages T1–T6 in each brain region shows that the stages with significant difference were concentrated in stages T2, T3 and T4. The frontal region as well as the occipital region in stage T5, and the parietal-occipital region in driving stages T5 and T6 also showed significant differences.

(b) Shortest Path Length Analysis

**Figure 6:**
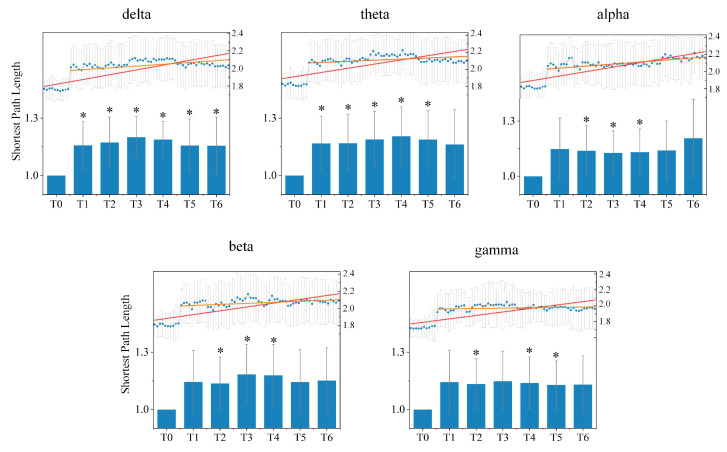
The average shortest path length of the directed brain network under five different rhythms in stages T0–T6 for all subjects. Histograms represent the relative changes from resting stage T0. The blue points in the histogram are the average shortest path lengths calculated from all sliding windows in one minute. The red line is the fitting line for all blue points over driving stages T0–T6, and the orange line is the fitting line for all blue points over driving stages T1–T6. * indicates a significant difference compared to stage T0 (one-way ANOVA followed by FDR correction, p<0.05).

In order to reveal changes in the global ability of the directed brain network to integrate information during driving, we quantified the shortest path length between any pair of nodes for each epoch under different rhythms during different driving stages for all subjects. Figure 6 shows the average shortest path length of the directed brain network under five different rhythms during seven experimental stages for all subjects. Compared to stage T0, the average shortest path length in stages T1–T6 increased under all rhythms, which indicates that information transfer between different nodes or brain regions requires passage through more nodes, and the efficiency of information integration is consequently reduced. During driving, the average shortest path length began with an upward trend until stage T3 under the delta, beta and gamma rhythm, and until stage T4 under the theta rhythm; a downtrend then followed. Nevertheless, there was initially a slight decrease followed by an obvious increase under the alpha rhythm as time went on.

The statistical differences of the average shortest path length under different rhythms between the resting stage T0 and driving stages T1–T6 were tested. Compared to stage T0, there were significant differences from stage T0 for stages T1–T6 under the delta rhythm, stages T1–T5 under the theta rhythm, stages T2–T4 under the alpha and beta rhythms, and stages T2, T4 and T5 under the gamma rhythm.

(c) Global Efficiency Analysis

**Figure 7:**
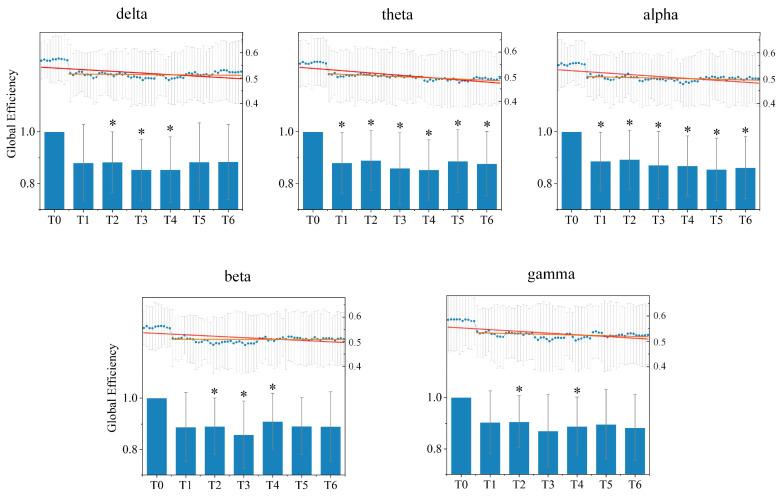
The average global efficiency of the directed brain network under five different rhythms in stages T0–T6 for all subjects. Histograms represent relative changes from the resting stage T0. The blue points in the histogram are the average global efficiencies calculated from all sliding windows in one minute. The red line is the fitting line for all blue points over the driving stages T0–T6, and the orange line is the fitting line for all blue points over the driving stages T1–T6. * indicates a significant difference compared to stage T0 (one-way ANOVA followed by FDR correction, p<0.05).

Next, we further explored changes in the global information integration ability of the directed brain network under different rhythms in seven stages T0–T6 for all subjects from the aspect of the global efficiency, as shown in Figure 7. A decrease in the average global efficiency appears in driving stages T1–T6 in comparison with the resting stage T0 under all rhythms. Moreover, for most rhythms except the alpha rhythm, the average global efficiency gradually declined to the minimum during driving stage T3 or T4, and then increased slightly, which implies that driving fatigue reduces the efficiency of information transmission in the directed brain network, in accordance with the results of the shortest path length analysis. Meanwhile, the average global efficiency under the alpha rhythm shows a continuous downward trend as driving time increased.

After statistical analysis, it could be found that the significant difference compared to the resting stage T0 existed for driving stages T1–T6 under the theta and alpha rhythm, for stages T2–T4 under the delta rhythm, for stages T2–T5 under the beta rhythm, and for stages T2 and T4 under the gamma rhythm.

### 4.3. Causal Flow Analysis

Based on the above results, significant differences were usually found between stage T4 and stage T0, such that stage T4 was selected to be representative of the driving fatigue state, while stage T0 represented the awake state for the causal flow analysis. Then, we analyzed the out-degree, in-degree and the causal flow of each node under different rhythms in order to find the key nodes that were significantly influenced by driving fatigue in terms of the causal information flow in the directed brain network.

**Figure 8:**
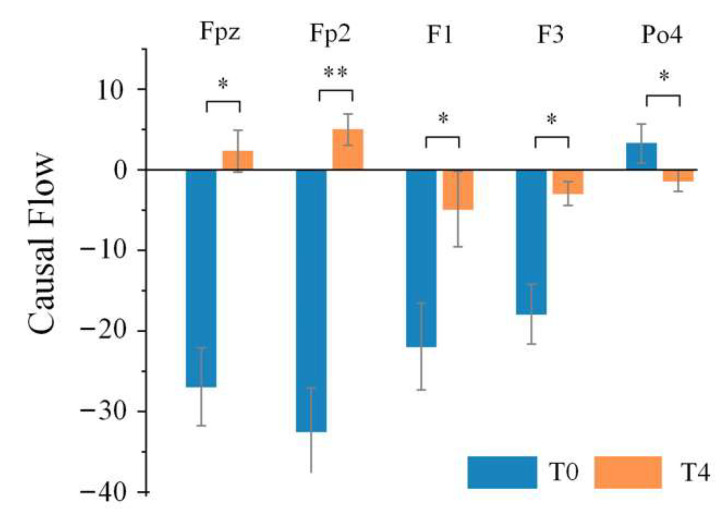
The average causal flow of key nodes that were significantly different between stage T0 and stage T4 under the theta rhythm for all subjects. * and ** indicate significant differences compared to resting stage T0 (one-way ANOVA followed by FDR correction; * stands for p<0.05 and ** stands for p<0.01 ).

Table 2 lists the electrodes with statistical differences in causal flow between stage T0 and stage T4 under different rhythms. Although the nodes with significant differences under different rhythms were not the same, they are mainly distributed in the anterior and posterior regions, and especially obvious under the theta rhythm. Moreover, it can be seen from Figure 8 that electrodes Fpz, Fp2, F1 and F3 in the anterior regions were important input sources in stage T0, while causal flow decreased at electrodes F1 and F3; electrodes Fpz and Fp2 became important output sources in stage T4. Besides, electrode Po4, located in the posterior regions, was an output source in stage T0, and then became an input source in stage T4. After calculating the causal flow metrics in stage T0 and stage T4 under different rhythms, only the theta rhythm, which contained the largest number of key nodes that were significantly different, was focused on for further studies on the effect of driving fatigue on information flow.

**Figure 9:**
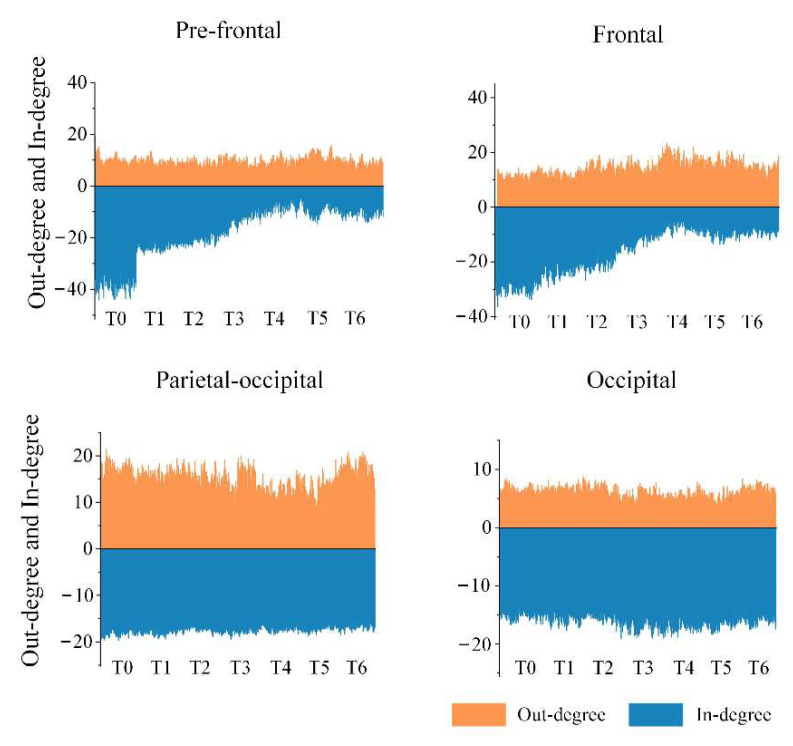
Evolutions of the average out-degree and the average in-degree over the important brain regions in stages T0–T6 under the theta rhythm.

In view of the above results, the important brain regions that specifically contained key nodes with significant differences were the pre-frontal, frontal, parietal-occipital and occipital regions in the anterior or posterior regions. We calculated the average of out-degree (or in-degree) over all electrodes in each important brain region under the theta rhythm, and the results are shown in Figure 9. It is apparent that the out-degree of the pre-frontal region did not change much with increased driving time, but the in-degree gradually decreased and appeared particularly obvious in stage T4. This suggests that the aggravation of fatigue may result in a reduction in information-receiving ability of the pre-frontal region. We also obtained a similar result for the frontal region regarding the in-degree, yet there was a slight increase in the out-degree, which implies that fatigue may induce an enhancement in information-sending ability of the frontal region to a certain extent. Moreover, a decline in the out-degree in the parietal-occipital region means a decrease in the information-sending ability, which is probably related to driving fatigue. Besides, for the occipital region, there was no significant change in the out-degree or the in-degree.

Furthermore, we also investigated the way brain regions communicate with each other as well as the effects of driving fatigue on communication. Next, the four important brain regions were considered as target regions, and the information flow between the target brain region and others was measured one-by-one under the theta rhythm in stages T0 and T4, according to the inflow or outflow of information. The following results show the changes in direct information transmission for different brain regions only, but without statistical support.

**Figure 10:**
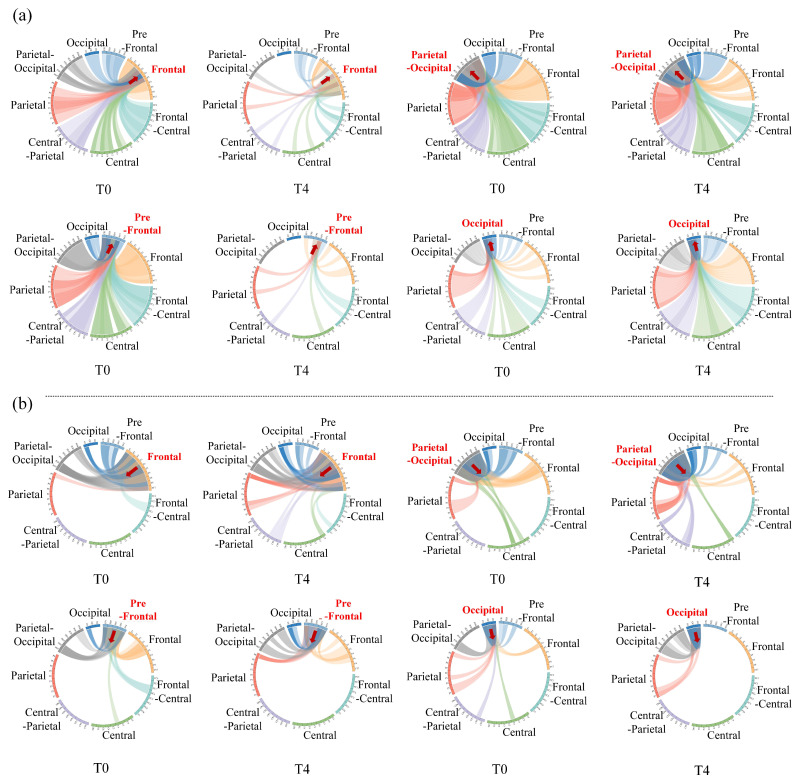
Chordal graph of the information flow between different brain regions in the average directed brain network under theta rhythm during stages T0 and T4. (**a**) Information inflow of the target brain region. (**b**) Information outflow of the target brain region.

Figure 10 intuitively illustrates the effect of driving fatigue on information flow between brain regions in the directed brain network. Figure 10a shows the information inflow to the target brain region. It can be seen that the inflow to the frontal (or pre-frontal) region from the central, central-parietal, parietal, parietal-occipital and occipital regions clearly decreased, suggesting that there was a significant reduction in information flow from the posterior regions to the anterior regions induced by driving fatigue. Besides, the parietal-occipital region is important for information inflow in both stages T0 and T4. However, the information inflow from other brain regions to the parietal-occipital region was slightly weakened by driving fatigue. At the same time, the information inflow from other brain regions to the occipital region was significantly enhanced. Therefore, under the conditions of driving fatigue, inflow to the occipital region from other posterior regions became an important brain region of information inflow, and this is mainly manifested in the transmission of information to the posterior regions. Figure 10b illustrates the information outflow of the target brain region. For the frontal and the prefrontal regions, outflow to the parietal-occipital and to the occipital regions was strengthened, which implies that driving fatigue enhanced information flow from anterior regions to posterior regions. Moreover, the outflow from the parietal-occipital region to the frontal and pre-frontal regions decreased, while the occipital region even disconnected with frontal regions at times, indicating that driving fatigue reduced the information flow from posterior regions to anterior regions. The above results fully demonstrate that fatigue driving significantly reduces the posterior region’s ability to receive information, while the anterior region’s ability of receiving information increases to a certain extent. Moreover, information transmission from the posterior regions to the anterior regions is significantly reduced, while information is mainly transmitted from anterior regions to posterior regions.

## 5. Discussion

During the past few years, many studies have focused on driving fatigue from the perspective of complex networks based on EEG signals. Some of these studies [11,17,21] combined machine learning to identify the brain states of the driver, which are practical for online fatigue monitoring. Meanwhile, this study’s method was an offline analysis. This paper aims to reveal the effect of driving fatigue on the brain’s information processing activities.

Our previous study [14] showed that based on the undirected brain network, under deepening fatigue the shortest path length significantly decreased, while the clustering coefficient and the number of functional connections significantly increased in the delta rhythm. However, considering that information flow within or between brain regions has direction, we used the directed brain network to analyze changes in brain dynamics during simulated driving. Directed brain networks can reflect whether there are connections between nodes, in addition to the directions of these connections.

Most studies established the brain complex network using scalp EEG signals directly. Although scalp EEG signals have high temporal resolution that can reflect rapid neural electrical activity, their spatial resolution is very low due to volume conduction and space smearing effects. Hence, if scalp EEG signals are directly adopted to construct a network, biases will exist in estimating connectivities between electrodes, especially for neighboring electrodes. Some studies adopted a connectivity index that is insensitive to the volume conduction effect, such as generalized partial directed coherence [20]. However, in this paper, we overcame the volume conduction effect by introducing a spherical spline interpolation Laplace algorithm to convert scalp EEG signals into current source density data, which could provide a clearer, reference-free, higher spatial resolution brain topography [25,42], and hence improve the estimation accuracy of connectivity [43].

The structure of a complex network is very sensitive to the threshold of connectivity strength. Different thresholds may result in different network structures. If a fixed threshold is set for all subjects, the individual differences between subjects will be neglected. There have been no criteria established for selecting thresholds thus far. However, there are several methods to determine the threshold, such as using mean values of the connectivity matrix [17], traversing all possible thresholds [11,14] and applying minimum spanning trees [18,44]. Optimal sparsity method is another method adopted in this paper that ensures that critical connections or edges are maintained [39].

We established a directed brain network by computing the directed transfer functions between pairwise electrodes, and furthermore studied evolutions and changes in small-world characteristics of these complex networks during fatigue driving, including the shortest path length, the clustering coefficient and the global efficiency. The results show that the average clustering coefficients for delta, theta and alpha rhythms increase regularly with increased driving time. The average path lengths of delta, theta, and alpha rhythms also increase regularly. However, the global efficiencies of delta and theta rhythms decrease regularly, showing a downward trend overall. It is suggested that local information integration ability enhances, while global integration efficiency declines under certain rhythms as a result of driving fatigue. There are also several related papers that studied driving fatigue based on directed brain networks. Dimtrakopoulos et al. [20] constructed a directed brain network that was based on generalized partial directed coherence for a simulated driving task, and found that the clustering coefficient as well as the characteristic path length under the theta rhythm have positive correlations with time. This is consistent with our results, and we furthermore found similar change trends in other rhythms, such as the delta and alpha rhythms. Kong et al. [24] established a directed brain network that was based on spectral Granger causality, and compared the drowsy stage with the alert stage for a driving task. They found that global efficiency during the drowsy stage was significantly lower than that during the alert stage in the delta and theta rhythms, similarly with our results. Meanwhile, the characteristic path length during the drowsy stage was significantly lower than that of the alert stage in the delta and theta frequency bands, a finding which deviates from our study. We may attribute this difference to electrode numbers, the network construction method used and the experimental task design.

Fatigue is a very complex brain state. Cognition can be impaired by fatigue that is induced by driving [45]. As the fatigue deepens during driving, both increases in the shortest path length and decreases in global efficiency indicate a decline in efficiency over the whole brain, while increases in the clustering coefficient denote better communication among nodes within a local brain region; this indicates an enhancement in local information integration ability of the brain to resist fatigue aggravation as well as brain efficiency reduction. In the later stages of driving fatigue, a slightly opposite trend was found for all these three brain network characteristics of fatigue driving compared with trends in the early and middle stages. This can be explained as self-adjustments in brain function to relieve the fatigue state slightly; nevertheless, there are still significant differences compared to the awake state.

In order to find out which critical electrodes were most affected by driving fatigue in terms of information communication, causal flow analysis was subsequently performed under different rhythms. We found that most key electrodes were located in the frontal, prefrontal and occipital regions, and that the nodes Fpz, Fp2, F1, F3 and Po4 under theta rhythm were the most significant. Wang et al. [21] employed betweenness centrality to illustrate the important electrodes in the directed brain network; the identified important brain regions where the important electrodes were located were consistent with our results, even though their specific electrode selection differed slightly with ours. This was partly because they used much fewer electrodes in their study than ours, resulting in neglect of the influence of some unselected electrodes in the construction of their brain network. Kong et al. [24] also found electrodes with significant changes widely located in the prefrontal, parietal, posterior midline, frontal and central regions in all EEG rhythms.

We further investigated the effect of driving fatigue on changes in information communication between different brain regions. Significant changes were found in the pre-frontal, frontal, parietal-occipital and occipital regions in the fatigue state compared to the awake state under the theta rhythm. Moreover, the information-receiving ability of anterior regions was obviously deteriorated, while the ability of posterior regions to receive information improved to a certain extent under the fatigue state compared to the awake state. This finding is consistent with those of Dimitrackopoulos et al. [20], indicating that information flow is directed from the anterior towards the central and posterior areas, induced by fatigue in the theta rhythm. Chen et al. [11] also mentioned that there were significant differences in functional connectivity among brain regions between the alert and fatigue states, especially in connections between the frontal region and the parietal region, which weakened.

Mental fatigue often occurs in long-term monotonous cognitive activities; this weakens peoples’ perceptual and operational abilities, resulting in movement errors [20,21]. Under well-controlled experimental conditions, the fatigue driving-inducing paradigm in subjects is usually achieved through performing vigilance or sustained attention tasks [18,20,24]. In this paper, by designing a monotonous and boring long-term driving task, the cognitive abilities of subjects was reduced, resulting in decreases in vigilance and attention, and causing driving fatigue. Although different paradigms can be used to induce fatigue, it remains unclear whether the underlying neural mechanisms are the same.

## 6. Conclusions

In order to study the influence of fatigue on brain dynamics during fatigue driving, we designed a monotonous driving experiment that made subjects prone to fatigue, and sustainably collected EEG signals that could directly reflect the neural electrical activity of the brain. In considering the volume conduction effect in the brain, we employed current source density data derived from EEG source analysis for further analysis instead of directly using EEG signals; this approach was in contrast to methods used in existing studies. Given that information communication in the brain has direction, we constructed a directed brain network based on directed transfer function indexes between electrodes. Changes in properties of the directed brain network induced by driving fatigue were analyzed using complex network theory. Based on the clustering coefficient, the shortest path length, the global efficiency and the causal flow extracted from the directed brain network under different rhythms, we found that as driving time increases, local information integration abilities gradually increase while global information integration abilities gradually decrease; moreover, information transmission from the posterior regions to the anterior regions becomes more difficult under certain rhythms. Our results may be helpful in revealing the neural mechanisms of brain fatigue.

## Figures and Tables

**Table 1:** Average GCE values corresponding to different S during driving stages T0–T6 under the theta rhythm.

	Stage	T0	T1	T2	T3	T4	T5	T6
Sparsity								
0.2		0.2463	0.2382	0.2417	0.229	0.219	0.2268	0.2251
0.25		0.2641	0.2604	0.2623	0.2546	0.239	0.251	0.2544
0.3		***0.2704***	***0.2697***	***0.2702***	0.2658	0.252	***0.2624***	***0.2687***
0.35		0.2687	0.269	0.2671	***0.2671***	***0.2513***	0.2618	0.2622
0.4		0.2596	0.2609	0.257	0.2606	0.248	0.2563	0.2609
0.45		0.2472	0.2477	0.2441	0.2487	0.2394	0.2435	0.2556
0.5		0.2316	0.2314	0.2281	0.2328	0.2259	0.2282	0.2395
0.55		0.2137	0.213	0.2102	0.2144	0.2096	0.2104	0.2197
0.6		0.1933	0.1927	0.1909	0.1937	0.1896	0.1908	0.1977

The maximum value of each column is marked in bold and italics.

**Table 2:** Electrodes with statistical differences in causal flow between stage T0 and stage T4 under different rhythms.

	Rhythms	Delta	Theta	Alpha	Beta	Gamma
Brain Region						
Pre-frontal			Fpz, Fp2	Fp2		Fp2
Frontal		F1	F1, F3			
Parietal-occipital		Po4	Po4		Po2	
Occipital						P7

## Data Availability

Not applicable.
